# Portable rapid detection of maize chlorotic mottle virus using RT-RAA/CRISPR-Cas12a based lateral flow assay

**DOI:** 10.3389/fpls.2023.1088544

**Published:** 2023-03-03

**Authors:** Rong Lei, Ruirui Kuang, Xuanzi Peng, Zhiyuan Jiao, Zhenxing Zhao, Haolong Cong, Zaifeng Fan, Yongjiang Zhang

**Affiliations:** ^1^ Institute of Plant Inspection and Quarantine, Chinese Academy of Inspection and Quarantine, Beijing, China; ^2^ State Key Laboratory of Agro-biotechnology and MARA Key Laboratory of Surveillance and Management for Plant Quarantine Pests, College of Plant Protection, China Agricultural University, Beijing, China

**Keywords:** MCMV, Cas12a, RT-RAA, one-tube, lateral flow assay

## Abstract

**Introduction:**

Maize lethal necrosis seriously threatens maize production worldwide, which was caused by coinfection by maize chlorotic mottle virus (MCMV) and a potyvirid. To effectively control maize lethal necrosis, it is vital to develop a rapid, sensitive, and specific detection method for the early diagnosis of MCMV in host plant tissues.

**Methods:**

We established a rapid detection procedure by combining the one-step reverse-transcription recombinase-aided amplification (one-step RT-RAA) and CRISPR/Cas12a-based lateral flow assay in one tube (one-tube one-step RT-RAA/CRISPR-Cas12a), which can be implemented on a portable metal incubator at 37~42°C. Furthermore, the crude extract of total RNA from plant materials using alkaline-PEG buffer can be directly used as the template for one-step RT-RAA.

**Results:**

The developed one-tube one-step RT-RAA/CRISPR-Cas12a lateral flow assay can detect as low as 2.5 copies of the coat protein (CP) gene of MCMV and 0.96 pg of the total RNA extracted from MCMV infected maize leaves. Furthermore, the MCMV infected maize leaves at 5 dpi having no obvious symptoms was detected as weak positive.

**Discussion:**

The crude extraction method of total RNA from plant materials required no complicated device, and all the procedures could be implemented at room temperature and on a portable metal incubator, costing a total time of about 1h. The one-step RT-RAA reagents and CRISPR/Cas12a reagents can be lyophilized for easy storage and transportation of reagents, which makes this method more feasible for the filed detection. This method presents rapidness, robustness and on-site features in detecting viral RNA, and is a promising tool for the field application in minimally equipped laboratories.

## Introduction

Maize (*Zea mays* L.) is one of the most important cereal crops in the world. In 2011, a large outbreak of maize lethal necrosis disease (MLND) occurred in the Southern Rift Valley of Kenya (Wangai et al., 2012), caused up to 100% losses in maize yield, and affected the income of farmers (Redinbaugh and Pratt, 2009; Pratt et al., 2017). MLND is caused by a mixed infection of maize chlorotic mottle virus (MCMV) and a virus of the family *Potyviridae*, such as sugarcane mosaic virus (SCMV) or maize dwarf mosaic virus (MDMV). MCMV was first identified in Peru in 1971 (Castillo and Hebert, 1974), and found in Kansas/Nebraska, Argentina/Thailand, Mexico (Aguilera et al., 2019), Hawaii (Jiang et al., 1992), China (Xie et al., 2011; Wu et al., 2013), Kenya (Kusia et al., 2015), Rwanda (Adams et al., 2014), Ethiopia (Mahuku et al., 2015), Taiwan, China (Deng et al., 2014), Ecuador (Quito-Avila et al., 2016), Spain (Achon et al., 2017) and other countries/regions (Lukanda et al., 2014; Pratt et al., 2017). Since SCMV is worldwide distributed (Redinbaugh and Pratt, 2009), MCMV is the emerging critical virus driving MLND expansion. Therefore, rapid and sensitive detection of MCMV is pivotal to effective control and management of the disease.

MCMV is only member of the genus *Macblomovirus* in the family *Tombusviridae*, which is readily transmissible to its natural host maize by mechanical damage, beetles or thrips (Nault et al., 1978) and seeds (Jiang et al., 1992). Present detection methods for MCMV include enzyme-linked immunosorbent assay (ELISA) (Uyemoto, 1980; Wu et al., 2013; Fentahun et al., 2017), immuno-fluorescence (Nault et al., 1979), surface plasmon resonance-based biosensor (Zeng et al., 2013), RT-PCR (Kiarie et al., 2020), quantitative TaqMan RT-PCR (Zhang et al., 2011; Zhang et al., 2016; Bernardo et al., 2021), the next generation sequencing (Adams et al., 2013), reverse transcription loop-mediated isothermal amplification (RT-LAMP) (Liu et al., 2016; Chen et al., 2017), reverse transcription recombinase polymerase amplification (RT-RPA) (Jiao et al., 2019; Gao et al., 2021) and reverse transcription recombinase-aided amplification/Cas12a (RT-RAA/Cas12a)-based visual detection (Duan et al., 2022). The immunological assay, *e.g.* ELISA and immune-fluorescence, relies on the quality and specificity of the antibodies (Bernardo et al., 2021). Quantitative RT-PCR requires an expensive thermal cycler with fluorescence detector. RT-LAMP requires a relatively high isothermal temperature (60 ~ 65°C), which requires high-capacity battery in portable field test. RPA requires relative low temperature (37 ~ 42°C), proceeds fast to produce exponential amplification of nucleic acid in the presence of two primers (Lei et al., 2019; Li et al., 2019a).

CRISPR (Clustered Regularly Interspaced Short Palindromic Repeats) based diagnostic system has been used to detect various nucleic acids (Kellner et al., 2019; Fapohunda et al., 2022). CRISPR-associated proteins (Cas) cleave foreign nucleic acids under the guidance of crRNA (East-Seletsky et al., 2016). Cas12a, an RNA-guided DNA endonuclease, exhibits its non-specific cleavage of single stranded DNA (ssDNA) after recognizing target dsDNA (Chen et al., 2018; Swarts and Jinek, 2019). Combining the cleavage effect of Cas12a with isothermal amplification created versatile rapid and specific platform, such as DETECTR (DNA Endonuclease Targeted CRISPR Trans Reporter) with fluorescence readout (Chen et al., 2018), Cas12VDet (Cas12a-based Visual Detection) in a one-pot reaction (Wang et al., 2019), lateral flow strips for visual readout (Gootenberg et al., 2017; Myhrvold et al., 2018), colorimetric detection with AuNPs-DNA probe (Li et al., 2019b; Jiao et al., 2020; Yuan et al., 2020). Importantly, these CRISPR-based diagnostic tests without complex apparatus can offer analytical sensitivities better than or comparable to real-time PCR technique, thus are suitable for field diagnosis application. Recently a visual detection of MCMV based on two-step RT-RAA and Cas12a technique using a blue light as the excitation light has been reported (Duan et al., 2022). However, the cDNA preparation, step-by-step experimental operation and visual observation based on fluorescence are not suitable for the purpose of field detection.

In this study, we integrated one-step RT-RAA with Cas12a-based lateral flow assay in one tube to develop a rapid detection method for MCMV with high specificity and sensitivity. By adopting a fast RNA exaction method free of device at room temperature, the developed one-tube one-step RT-RAA/Cas12a method can be used to detect MCMV in plant samples. Furthermore, the RT-RAA and Cas12a reagents can be lyophilized for easy storage and transportation (Lei et al., 2022), making this portable and sensitive detection strategy more suitable for plant virus in field.

## Materials and methods

### Virus sources, virus inoculation and RNA extraction

The sources of MCMV and SCMV were kept in our laboratory. Both viruses were propagated on maize inbred line B73 and cv. Nongda 108 plants, which were grown in a growth chamber (28 °C 16h light and 22°C 8h night cycles) for virus propagation. The first true leaves of one-week-old maize seedlings were rub-inoculated with the homogenized MCMV- and SCMV- infected maize leaf tissues in 0.01 M phosphate buffer [0.01 M KH_2_PO_4_: 0.01 M Na_2_HPO_4 =_ 49: 51 (v/v), pH 7.0] at a ratio of 1:10 (g/mL). The systemically infected leaves were harvested at about 10 days post inoculation (dpi). The total RNA of cucumber mosaic virus (CMV), tobacco mosaic virus (TMV), tomato ringspot virus virus (TRSV), tomato black ring virus (TBRV) were stored in our laboratory.

Extraction of total RNA from maize leaf tissue (~100 mg) was performed using *EasyPure* ^®^ RNA Purification Kit (TransGen, Beijing, China) according to the manufacturer’s instructions. The extracted nucleic acid was dissolved in 50 μL nuclease-free water and stored at -80°C prior to testing. Total RNA was quantified using a BioTek Epoch spectrophotometer (BioTek, Vermont, USA).

The crude extracts of total RNA from plant materials was extracted with modified alkaline polyethylene glycol (PEG) extraction method (Chomczynski and Rymaszewski, 2006; Huang et al., 2013; Silva et al., 2018). In brief, the fresh leaf samples (about ϕ 8 mm size) were punched with the lids of 1.5 mL tubes, crushed with quartz sands using a plastic pestle, immersed in 100 μL of freshly prepared alkaline-PEG buffer [6% PEG 200 (Solarbio, Beijing, China) with 20 mM NaOH] (Huang et al., 2013; Silva et al., 2018), and incubated at room temperature for 4 min. The plant extract supernatants were tested immediately or kept on ice until further use.

### RT-RAA primers, fluorescent probe and crRNA design

**Figure 1:**
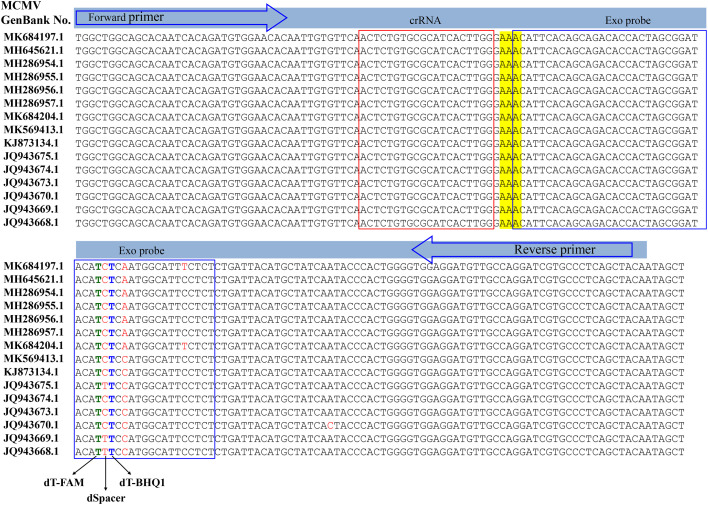
Design of the primer set and crRNA in the coat protein gene of MCMV.

RT-RAA primers and real time fluorescent probe were designed from the conserved coat protein region of MCMV following multiple sequence alignment of the available virus sequences in the GenBank database (**Figure 1**). The primers and probe were BLASTed against the GenBank to exclude other plant viruses, including SCMV, cucumber mosaic virus (CMV), tobacco mosaic virus (TMV), tomato ringspot virus (TRSV), tomato black ring virus (TBRV). The forward primer MCMV-F (30 nt) and reverse primer (33 nt) (**Table 1**) produced target amplicons of 116 bp. The real-time fluorescent probe consists of an oligonucleotide with homology to the target amplicon that contains a dSpacer which replaces a nucleotide in the target sequence flanked by a dT-FAM (fluorophore) and corresponding dT-BHQ1 (quencher). In addition, C3-spacer as a blocker was labelled at the 3’-end to prevent polymerase extension from the 3’-terminus. When the target amplicon was produced, this fluorescent probe is cleaved by the *E*. coli exonuclease III at the abasic site to separate the fluorophore “FAM” from the quencher “BHQ1” and generate an extensible 3’-OH group for polymerization, thus generating fluorescent signals. All primers and probe were synthesized by Sangon Biotech (Shanghai, China).

**Table 1:** Oligonucleotide sequences used in this study.

Oligonucleotide	Sequences (5’-3’)	Ref.
MCMV-F	TGGCTGGCAGCACAATCACAGATGTGGAACA	This study
MCMV-R	CTATTGTAG CTGAGGGCAC GATCCTGGCA ACAT	
MCMV-exoP	ACATTCACAGCAGACACCACTAGCGGATACA/iFAMdT//idSpace//iBHQ1dT/CMATGGCATTTCTCT-C3 spacer	
MCMV-crRNA	UAAUUUCUACUCUUGUAGAUCCAAGUGAUGCGCACAGAGU	
FQ reporter	FAM/TTATT/BHQ1	
LF reporter (5 nt)	FAM/TTATT/biotin	
LF reporter (20 nt)	FAM/TTTTTTTTTTTTTTTTTTTT/biotin (FAM/T_20_/biotin)	
MCMV-PF1	5’-TTATGGCGACCCTACCTTCAAT-3’	(Zhang et al., 2016)
MCMV-PR1	5’-ACCACCGTCATCTTCCGTTC-3’	
MCMV-probe	FAM-CACGCATTTTGGAAGCTGTGGTGGC-BHQ1	

The crRNA was designed to recognize a site specific to MCMV genome and to be homologous to a region within the amplicon between the RT-RAA primer pair binding sequences. The Cas12a/crRNAs recognize a 20-bp target sequence adjacent to a PAM site (TTTN or NAAA) site and were designed from the highly conserved region of each virus as found in the GenBank (NCBI). There was one PAM site (GAAA) in the RT-RAA amplicons of MCMV, whose reverse complement sequence is TTTC, so the corresponding reverse and complementary sequence was reversely transcribed as the DNA template for crRNA. The DNA template for crRNA was further checked its specificity by BLAST against the GenBank. The DNA sequences and modifications are outlined in **Table 1**. Fluorescent quencher reporter (FQ reporter: FAM-TTATT-BHQ1), lateral flow assay reporter (LF reporter: FAM-T_20_-biotin) and crRNA were synthesized by General BioL. (Anhui, China).

### One-step RT-RAA reaction of viral RNA

The real-time fluorescent detection of the viral RNA was performed with fluorescent RT-RAA kits according to the instructions of the RT-RAA fluorescent kit (Cat No. F00R01, Jiangsu Qitian Gene Biotechnology Co., Ltd., Jiangsu, China). Briefly, a master mix containing 25.0 μL of supplied rehydration buffer, 10.0 μL of DNase/RNase-free deionized water (Cat. No. RT121, Tiangen, Beijing, China), 2.4 μL of forward primer (10 μM), 2.4 μL of reverse primer (10 μM), and 2.5 μL of magnesium acetate (280 mM) was added to a RT-RAA pellet to dissolved the enzyme. And 2 μL of extracted RNA was added and mixed. Next, 2.5 μL of magnesium acetate (280 mM) was added on the tube lid, which was mixed with the RT-RAA reaction buffer with centrifugation. The Eppendorf tubes were put in a LightCycler 480 (Roche, USA) to record the emitted fluorescence signals. The excited and emitted wavelength are 488/520 nm, respectively. RNA extracted from healthy maize leaves and DNase/RNase-free deionized water were included as the negative control alongside tested samples.

The pre-amplification of the viral RNA for CRISPR/Cas12a detection was performed with RT-RAA kits according to the instructions of the RT-RAA basic kit (Cat. No. B00R00, Jiangsu Qitian Gene Biotechnology Co., Ltd., Jiangsu, China) with modifications. Briefly, a master mix containing 25.0 μL of supplied rehydration buffer, 10.0 μL of DNase/RNase-free deionized water (Cat. No. RT121, Tiangen, Beijing, China), 2.4 μL of forward primer (10 μM), 2.4 μL of reverse primer (10 μM), and 2.5 uL of magnesium acetate (280 mM) was added to a RT-RAA pellet to dissolved the enzyme, A reconstituted RT-RAA reaction aliquot of 9.0 μL was transferred to new PCR tubes, then 2 μL of extracted RNA was added to each reaction, and mixed by carefully pipetting up and down. The reactions were incubated at 39°C on a self-developed thermal block for 15-30 min. RNA extracted from healthy maize leaves and DNase/RNase-free deionized water were included as the negative control alongside tested samples.

### CRISPR/Cas12a based detection for one-step RT-RAA amplicons

The Cas12a-mediated fluorescent detection contained 1×NEBuffer 3.1 (Cat. No. B7203, New England Biolabs, Ipswich, MA, USA), 0.1 μM of EnGenLba Cas12a (Cat. No. M0653T, New England Biolabs, Ipswich, MA, USA), 0.12 μM of crRNA, 0.1 μM FQ reporter, 8U of RNase inhibitor (Cat. No. NG209, Tiangen, Beijing, China), 2.0 mM DTT (Cat. No. 43816, Sigma Aldrich, St. Louis, MO, USA) and 1 μL RT-RAA amplicons in 20 μL reaction volume. The reaction was performed at 37 °C in a LightCycler 480 (Roche, USA) to record the emitted fluorescence signals. The excited and emitted wavelength are 488/520 nm, respectively.

The Cas12a-mediated lateral flow assay contained 1×NEBuffer3.1 (New England Biolabs, Ipswich, MA, USA), 0.1 μM of EnGenLba Cas12a (New England Biolabs, Ipswich, MA, USA), 0.12 μM of crRNA, 1.0 μM LF reporter, 8U of RNase inhibitor (Tiangen, Beijing, China), 2.0 mM DTT (Sigma Aldrich, St. Louis, MO, USA) and 1 μL RT-RAA amplicons in 20 μL reaction volume. The reaction was performed at 37 °C in an incubator for 30 min and the products were detected with lateral flow strips (Cat. No. JY0301, Tiosbio Biotechnology Co, Ltd., Beijing, China).

### One-tube one-step RT-RAA/Cas12a based lateral flow detection

A master mix containing 25.0 μL of supplied rehydration buffer, 6.0 μL of nuclease free water, 4.0 μL LF reporter (10 μM), 2.4 μL of forward primer (10 μM), 2.4 μL of reverse primer (10 μM) was added to an RT-RAA pellet to dissolve the enzyme, followed by the addition of 2.5 uL of magnesium acetate (280 mM), then 9 μL of the reconstituted RT-RAA reaction buffer was distributed to the bottom of a new PCR tube. Meanwhile, the Cas12a reaction buffer containing 7.5 μL NEBuffer 3.1 (10×), 2.5 μL EnGenLba Cas12a (5 μM), 2.5 μL DTT (0.1 M), 2.5 μL RNase inhibitor (40U) and 5.0 μL crRNA (5 μM) was distributed into 4 μL aliquots, which was added on the PCR tube lid. If a larger number of reactions are needed, the master mix volume and pellets are scaled up accordingly. One microliter RNA sample was added into the bottom of the PCR tube, mixed by carefully pipetting up and down, then the PCR tubes were gently closed and put on an incubator at 37°C for 20 min. Subsequently, the CRISPR reagents pre-placed on the PCR tube lid were mixed with the RPA reaction buffer by inverting and centrifuging the tube, and incubated at 37°C for another 20 min. When the FQ reporter was used, the PCR tubes were put in a portable fluorimeter or real-time PCR instrument. When the LF reporter was used, the PCR tubes were put in an incubator and lateral flow strip was directly inserted into the reaction buffer after 85 μL water was added.

### Evaluation of sensitivity for fluorescent RT-RAA assay and RT-RAA/Cas12a based detection

Ten-fold serial dilutions of total RNA (96.0 ng/μL) and plasmid genomic DNA containing the MCMV coat protein gene (2.5×10^7^ copies) were used to evaluate the detection sensitivity of the real-time fluorescent RT-RAA assay and one-tube one-step RT-RAA/CRISPR-Cas12a detection in triplicate. The previously reported primers (MCMV-PF1/MCMV-PR1) and TaqMan probe (MCMV-Probe) (**Table 1**) of the TaqMan real-time RT-PCR method (Zhang et al., 2016) was employed as a control. The TaqMan real-time RT-PCR amplification was performed in a 20.0 μL of reaction volume containing 10 μL of 2×*PerfectStart*™ Probe One-Step qPCR SuperMix (Cat. AQ221, TransGen, Beijing, China), 0.4 μL of *TransScript* ^®^II Probe One-step RT/RI Enzyme Mix (Cat. AQ221, TransGen, Beijing, China), 0.4 μL of MCMV-PF1 (10 μM), 0.4 μL of MCMV-PR1 (10 μM), 1 μL of MCMV-probe (1 μM), 6.8 μL of RNase-free and 1.0 μL of RNA, were run on a LightCycler 480 (Roche, USA).

### Detection of MCMV in maize leaves

MCMV inoculated maize leaves were harvested at 5, 7 and 12 dpi, and the fresh maize leaves were punched with the lids of 1.5 mL tubes, and the crude extracts of total RNA from plant materials were prepared using freshly prepared alkaline-PEG buffer. One microliter of the supernatant was detected using this one-tube one-step RT-RAA/Cas12a assay. Three biological repeats were tested for each sample at different dpi.

## Results

### Strategy for portable detection of plant virus

**Figure 2:**
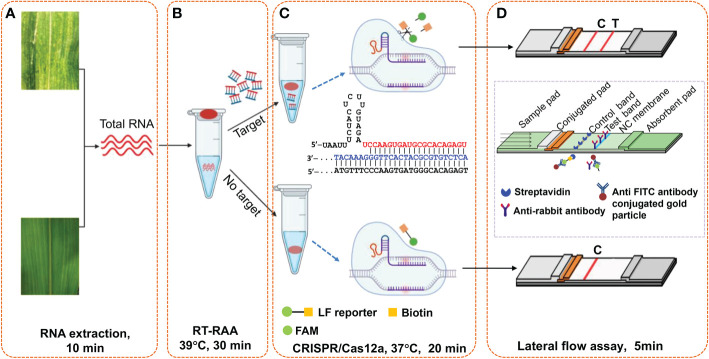
Schematic illustration of Cas12a-based one-tube plant viral RNA detection platform. **(A)** Total RNA extraction from plant tissue, **(B)** RT-RAA pre-amplification, **(C)** Cas12a/crRNA cleavage assay, and **(D)** visual readout of lateral flow strips. Total RNA was extracted from plant tissues with extraction kits or PEG-NaOH reagents. Cas12a/crRNA reagent mixture containing crRNA, cas12a enzyme and reaction buffer were added on the tube lid, which was shaken down to mix with the RT-RAA reaction buffer after the RT-RAA pre-amplification, followed by further incubation at 37°C for another 20 min. The lateral flow strips assay is complete in 5 min, and the bands can be immediately visually observed.

As illustrated in **Figure 2**, this Cas12a-based one-tube plant viral RNA detection platform using lateral flow strips integrates (A) RNA extraction, (B) RT-RAA pre-amplification of plant viral RNA, (C) sequence-specific recognition of amplicons and non-specific cleavage of LF reporters by Cas12a/crRNA, and (D) visual detection of cleaved product from LF reporter. Both RT-RAA amplification reaction buffer and Cas12a/crRNA buffer were loaded into the same tube before the reaction started, and the Cas12a/crRNA buffer was mixed with RT-RAA pre-amplification reaction buffer by shaking operation after the RT-RAA finished. When the Cas12a reaction ended, water was added into the tube to dilute the reaction buffer, and a lateral flow strip was inserted to detect the cleaved products of LF reporters. As shown in **Figure 2C**, the cleavage ability of CRISPR/Cas12 complex is activated when the crRNA specifically complements with the target DNA amplicons, and the LF reporters modified with both FAM- and biotin-group was cleaved to produce molecules with free FAM- or biotin-group. When the lateral flow strip contacts with the diluted Cas12a reaction buffer, the FAM-group in the buffer conjugates with the anti-FITC antibody on the surface of AuNPs immobilized at the conjugation pad. For negative sample, the LF reporter is intact, and the FAM-AuNPs conjugates have biotin-group, which would be captured by the streptavidin immobilized on the control area to form the control band. For positive sample, the FAM-AuNPs conjugate passes through the control area due to the lack of biotin group, arriving at the test area to form positive band (**Figure 2D**). Therefore, for the negative sample, only control band appears, while both test band and control band appear for the positive control, or only test band for the strong positive control. The whole experiment including both RT-RAA and Cas12a reaction can be performed on a home-made portable incubator between 37~42°C. By employing the fast plant RNA extraction procedure, the total time from sample preparation to results readout is about 1 h.

### Specific detection of MCMV

**Figure 3:**
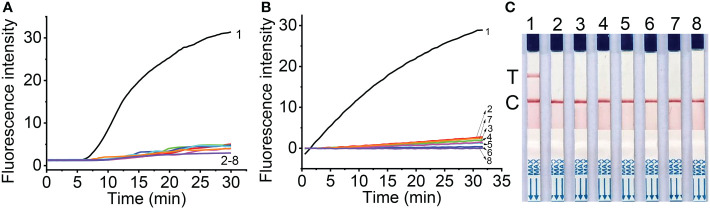
Specificity of real-time fluorescent RT-RAA **(A)**, Cas12a mediated fluorescent detection **(B)** and lateral flow assay **(C)** of RT-RAA pre-amplification products from total RNA. 1, Maize chlorotic mottle virus (MCMV); 2, Sugarcane mosaic virus (SCMV); 3, Cucumber mosaic virus (CMV); 4, Tobacco mosaic virus (TMV); 5, Tomato ringspot virus (TRSV); 6, Tomato black ring virus (TBRV); 7, healthy maize leaves; 8, DNase/RNase-free deionized water.

The specificity of RT-RAA primers was first evaluated using the real-time fluorescent RT-RAA assay. As shown in **Figure 1**, the forward and reverse primers covered the conserved sequences of all the MCMV strains, while the sequences after the “dSpacer” group have one or two different bases. The real-time fluorescence signals showed that only RNA extracted from MCMV infected maize leaves produced obvious fluorescence intensity, but no intense fluorescence intensity was produced by the total RNA extracted from SCMV- infected maize leaves, healthy maize leaves and other plant virus infected plant leaves, which demonstrated that the RT-RAA primers are specific for MCMV (**Figure 3A**).

The specificity of RT-RAA primers and crRNA for RT-RAA/CRISPR-Cas12a detection were tested with the CRISPR-Cas12a based fluorescent detection and lateral flow assay. The results showed that other plant viruses, such as SCMV, CMV, TMV, TRV, TBRV produced very low fluorescence signals (**Figure 3B**), while MCMV produced strong fluorescence signals, indicating that the primers and crRNA have good specificity for MCMV. The lateral flow strips results demonstrated that only MCMV showed up on the test band, which was consistent with that of fluorescent detection (**Figure 3C**).

### Optimization of Cas12a/crRNA-mediated lateral flow assay of MCMV RNA

**Figure 4:**
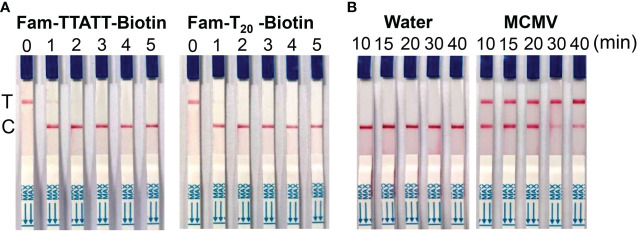
**(A)** Optimization of LF reporter concentration for lateral flow strips detection. 0, 0 nM; 1, 50 nM; 2, 100 nM; 3, 200 nM; 4, 500 nM; 5, 1 μM in the final detection buffer; **(B)** Optimization of CRISPR/Cas12a reaction time.

In this study, we tested 5 nt and 20 nt ssDNA modified with FAM- and biotin-group with different concentration to check the effect of LF reporter on the control and test bands. The results showed that 100 nM LF reporter (20 nt) in the final detection buffer is enough to avoid the false positive phenomenon (**Figure 4A**). To optimize the CRISPR/Cas12 reaction time, the lateral flow strips results were compared using MCMV RNA (positive control) and DNase/RNase-free deionized water (negative control) as the template for RT-RAA. The results showed that MCMV RNA as the template could generate obvious test bands with 10 min CRISPR/Cas12a reaction time, while negative control produced no test bands (**Figure 4B**). Although the intensity of test band became darker with the increased CRISPR reaction time, long reaction time may generate non-specific products, and is not favorable for rapid detection. Therefore, 20-30 min is selected as the subsequent Cas12a/crRNA reaction time.

### One-tube one-step RT-RAA/CRISPR-Cas12a lateral flow strip detection

**Figure 5:**
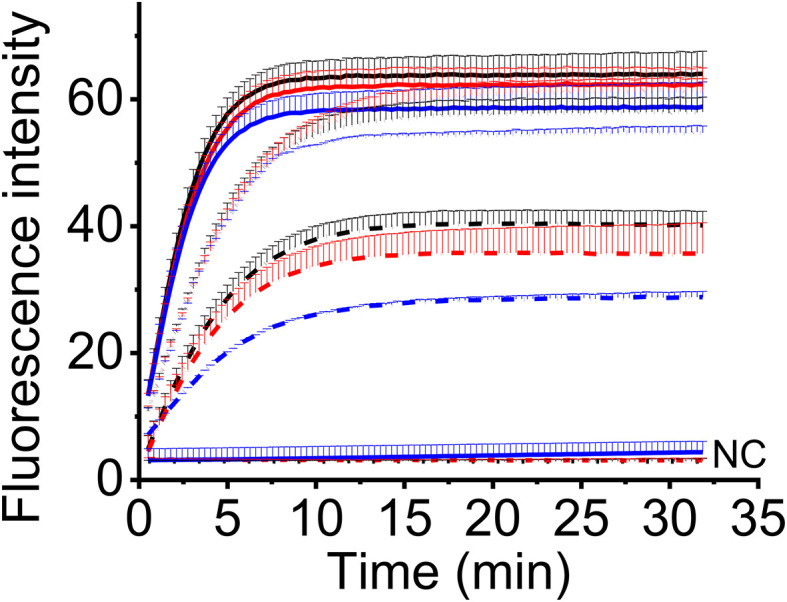
The real-time fluorescent intensity of different RT-RAA/CRISPR-Cas12a operation for different MCMV RNA amount. Black line, one-step operation; red line, two-step operation (10 μL RT-RAA buffer/10 μL CRISPR buffer); blue line, two-step operation (2 μL RT-RAA buffer/18 μL CRISPR buffer). Solid lines indicate the 9.6 ng total RNA; short dot lines indicate 9.6 × 10^-3^ ng total RNA; short dash dot lines indicate 9.6 × 10^-4^ ng total RNA. DNase/RNase-free deionized water was used as the negative control (NC).

To make this method feasible for the field detection, we adopted a one-tube strategy to avoid the step-by-step (two-step) experimental operation. In this strategy, one-step RT-RAA reagents and CRISPR-Cas12a reagents were put in one tube before the addition of sample RNA, so no lid opening was required during the experiment. To compare the detection efficiency of two-step and one-tube operation, three levels of MCMV RNA were tested using two-step way (10 μL RT-RAA reaction buffer was mixed with 10 μL CRISPR reaction buffer and 2 μL RT-RAA reaction buffer was mixed with 18 μL CRISPR reaction buffer) and one-tube way. There is no big difference amongst the three ways when high amount of MCMV RNA (9.6 ng and 9.6 × 10^-3^ ng) was used (**Figure 5**). When lower MCMV RNA was detected, the one-step operation generated the highest fluorescence signal, while the two-step (2 μL RT-RAA buffer was mixed with 18 μL CRISPR buffer) produced the lowest. Therefore, one-tube operation is a good alternative for the combined one-step RT-RAA with CRISPR-Cas12a detection system.

### Sensitivity assessment of the real-time fluorescent RT-RAA

**Figure 6:**
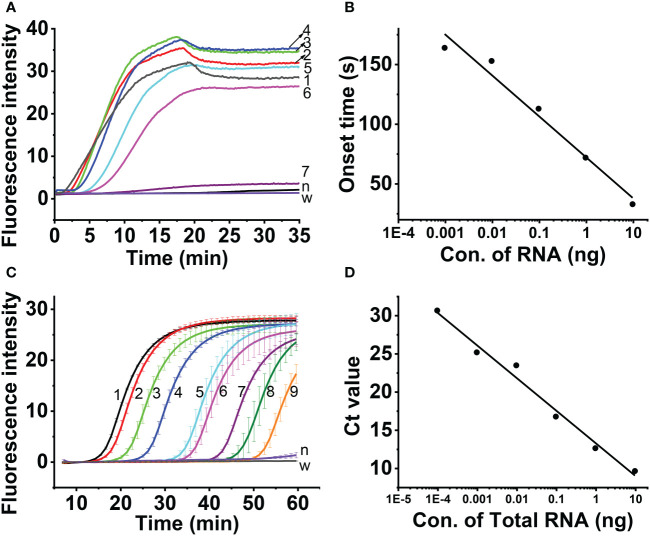
Sensitivity assessment of real-time one-step RT-RAA **(A, B)** and TaqMan real time RT-PCR **(C, D)**, Lines 1 ~ 8 indicated the amount of total RNA. 1, 96 ng; 2, 9.6 ng; 3, 0.96 ng; 4, 96 pg; 5, 9.6 pg; 6, 0.96 pg; 7, 96 fg; 8, 9.6 fg; 9, 0.96 fg; n, healthy maize plant; w, water.

The results showed that the real-time fluorescent RT-RAA assay could detect the total RNA down to 10^- 6^ dilution of total RNA (9.6× 10^-5^ng), whose weak fluorescence signal could be detected (**Figure 6A**). Since the RNA was exponentially amplified by the RAA technique, the onset time of amplification was plotted against the log concentration of the total RNA. The highest RNA concentration (96.0 ng) and the lowest RNA concentration (9.6 × 10^-5^ ng) were excluded out of the linear range, and the concentration range from 9.6 ng to 9.6 × 10^-4^ ng was linearly fitted with a R^2^ value of 0.963 (**Figure 6B**).

Aa comparison, the TaqMan real-time RT-PCR detection results showed that although the total RNA down to 10^-8^ dilution of (9.6 × 10^-7^ng) generated fluorescence signal (**Figure 6C**), the Ct value of the 3 replicas is 40, 37.59 and 38.37, respectively. The 10^-7^ dilution of total RNA (9.6×10^-6^ng) generated fluorescence signal, and the Ct value of the 3 replicas 32.99, 35.29 and 34.94, respectively. The 10^-6^ dilution of total RNA (9.6 × 10^-5^ng) generated fluorescence signal, and the Ct value of the 3 replicas 30.69, 30.8 and 30.55, respectively. Since Ct value of 35 was regarded as the critical value, the linearity was evaluated using the total RNA from 9.6 ng to 9.6 × 10^-5^ ng, and the Ct value was fitted against Log (total RNA) with a R^2^ value of 0.98 (**Figure 6D**).

### Sensitivity assessment of one-tube one-step RT-RAA/CRISPR-Cas12a detection

**Figure 7:**
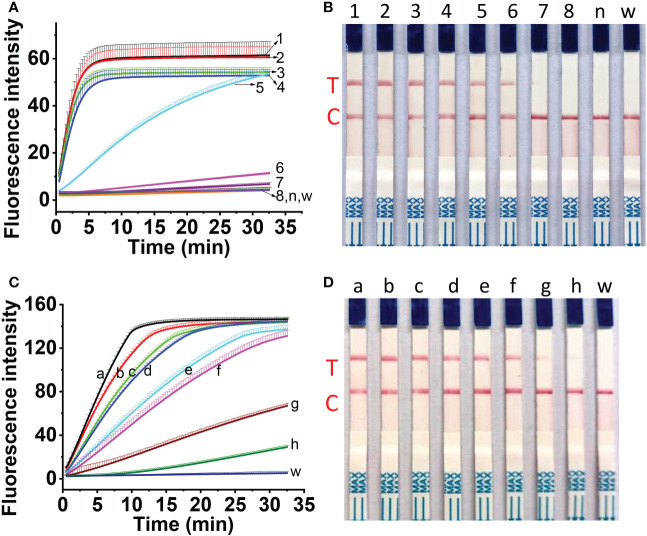
Sensitivity assessment of one-tube one-step RT-RAA/Cas12a-based detection for 10-fold diluted total RNA **(A, B)** and for 10-fold diluted plasmid DNA containing coat protein gene **(C, D)**. **(A, B)** No.1 ~ 8 indicated the amount of total RNA. 1, 96 ng; 2, 9.6 ng; 3, 0.96 ng; 4, 96 pg; 5, 9.6 pg; 6, 0.96 pg; 7, 96 fg; 8, 9.6 fg; n, RNA from healthy maize leaves; w, water. **(C, D)** a ~ h indicated the copies of plasmid containing the coat protein gene of MCMV. a, 2.5 × 10^7^ copies; b, 2.5×10^6^ copies; c, 2.5×10^5^ copies; d, 2.5×10^4^ copies; e, 2.5×10^3^ copies; f, 2.5×10^2^ copies; g, 2.5×10^1^ copies; h, 2.5 copies; w, water.

The sensitivity of one-tube one-step RT-RAA/Cas12a detection for MCMV was evaluated using the ten-fold serially diluted RNA as the template. The results showed that 10^-6^ dilution of total RNA (96 fg) can be detected using the fluorescent detection (**Figure 7A**), and 10^-5^ dilution of total RNA (0.96 pg) can be obviously detected using the lateral flow assay (**Figure 7B**). Since one-step RT-RAA/Cas12a involved exponential amplification and enzyme/nucleic acid reaction, there is no good mathematic mode to explain the relation between the fluorescence signal and RNA concentration. To determine the absolute sensitivity, plasmid genomic DNA containing MCMV coat protein gene ranging from 2.5 to 2.5×10^7^ DNA copies were tested. Both the fluorescent detection and lateral flow assay could detect as low as 2.5 copies (**Figures 7C, D**).

### Detection of MCMV in maize leaves

**Figure 8:**
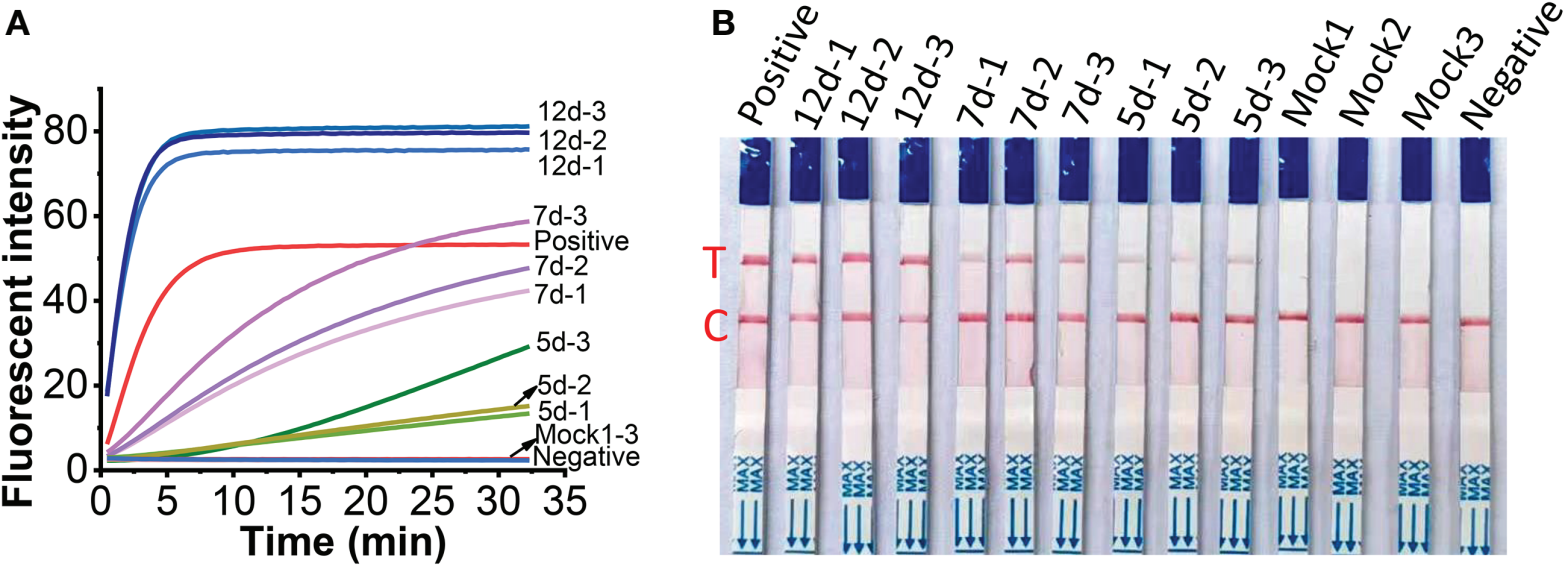
Detection of crude extracts of total RNA from MCMV inoculated maize leaves at different day post inoculation (dpi). **(A)**, one-tube one-step RT-RAA/Cas12a fluorescent detection; **(B)**, one-tube one-step RT-RAA/Cas12a lateral flow assay. Mock1~3, the mock inoculated maize leaves; 5d-1~5d-2, the MCMV inoculated maize leaves at 5 dpi; 7d-1~7d~3, the MCMV inoculated maize leaves at 7 dpi; 12d-1~12d-3, the MCMV inoculated maize leaves at 12 dpi.

To check the feasibility of the developed one-tube one-step RT-RAA/Cas12a lateral flow assay, MCMV were tested in inoculated maize leaves at different days post-inoculation (dpi). The crude extracts of total RNA from MCMV inoculated maize leaves produced obvious fluorescence signals, whose intensity increased with longer infection time from 5 to 12 days (**Figure 8A**), while there was no fluorescence signal resulted from the mock inoculated maize leaves (**Figure 8A**, Line Mock 1-3). Accordingly, the one-tube one-step RT-RAA/Cas12a based lateral flow assay showed that no test band was detected from the crude extracts of total RNA from mock inoculated healthy maize leaves, but the MCMV inoculated maize leaves at 5 dpi was detected as weak positive (**Figure 8B**). Results of both fluorescent detection and lateral flow assay demonstrated that the amount of MCMV in maize leaves increased with the longer infection time, indicating that this one-tube one-step RT-RAA/CRISPR-Cas12a can be used to detect the field plant sample.

## Discussion

The positive-sense RNA genome of MCMV (4437 nucleotides) encodes six proteins, i.e. P32 (32 kDa protein), RNA dependent RNA polymerase (P50 and P111), P31 (31 kDa protein), P7 (7 kDa protein), and coat protein (25 kDa) (Nutter et al., 1989; Stenger and French, 2008; Scheets, 2016). Coat protein gene is the most used target for MCMV detection (Zhang et al., 2011; Chen et al., 2017; Jiao et al., 2019; Duan et al., 2022). The P32 gene (Gene ID: 26522936) was also used to detect MCMV (Zhang et al., 2016). Integral coat protein is indispensable for the cell-to-cell movement of MCMV virions in plants (Scheets, 2016). Therefore, the conserved sequences of coat protein region are ideal targets for the detection of MCMV. In this study, the RT-RAA primers were designed based on the coat protein genes of MCMV isolates whose sequences are available in the GenBank database. The primers cover the most conserved sequences of all the isolates as possible. Except of the primers for RT-RAA, the crRNA design has played an essential role in the sensitivity of Cas12-based pathogen detection (Roy and Kirchner, 2000; Chen et al., 2018). It is critical to establish a single crRNA strategy that monitors most MCMV strains *via* the Cas12a-based detection. The recognition of dsDNA by Cas12a requires the target DNA strands to contain the protospacer adjacent motif (PAM) sequence of TTTN or AAAN (Chen et al., 2018; Gootenberg et al., 2018; Yan et al., 2019; Karvelis et al., 2020). There was one PAM site (GAAA) in the RT-RAA amplicons of MCMV, whose reverse complement sequence is TTTC, so the corresponding reverse and complmentary sequence was reversely transcribed as the DNA template for crRNA. The further check of the specifity by BLAST against GenBank showed that the DNA template for crRNA was specific to MCMV coat protein gene.

Developing a portable and rapid detection approach is a practical requirement for the monitoring of MCMV in farmland. Since gel electrophoresis is unsuitable for diagnosis of plant pathogen in the field (Jiao et al., 2019; Gao et al., 2021), we turned our attention to the lateral flow assay, one of the most convenient portable field detection techniques, which have been widely used for pathogen detection (Corstjens et al., 2001; Mao et al., 2009; Wang et al., 2020). Although a number of laboratory and commercial assays are available for detection of MCMV in maize plant, the rapid and accurate detection applicable for field detection was still limited. LAMP is an isothermal nucleic acid amplification technique, but it requires four primers, complex primer design and a reaction temperature as high as 63˚C, which is difficult to achieve in remote field. Recombinase-aided amplification (RAA) or Recombinase polymerization amplification (RPA) adopts three key proteins, i.e. recombinase, recombinase loading factor and single-stranded binding protein in reaction buffer, and run between 37~42˚C (Li et al., 2019a). As we know, almost all the applications of RPA or RAA for MCMV RNA detection adopted two-step operation, that is, total RNA was firstly transcribed to cDNA, then the cDNA was amplified in RPA or RAA reaction system and detected by electrophoresis (Jiao et al., 2019; Gao et al., 2021), or by Cas12a based visual detection (Duan et al., 2022). The synthesis of cDNA needs additional time from 30 min to 60 min at a temperature between 37˚C and 42˚C. To save the time and simplify the experimental operation, we adopted the one-step RT-RAA procedure which includes the reverse transcriptase and RAA enzymes in one reaction buffer, thus the reverse transcription and amplification could carry out simultaneously. Furthermore, to reduce the reliance on equipment, simplify the operations and avoid contamination, we established a one-tube one-step RT-RAA/Cas12a lateral flow assay for MCMV in maize, requiring minimum equipment, such as pipettes, reagent tubes, a portable thermal block, and lateral flow strips. The feasibility of one-pot detection including RPA and CRISPR-Cas12a reaction has been assessed in our previous work (Lei et al., 2022), the results (**Figures 5**, **8**) demonstrated that one-tube one-step RAA/CRISPR-Cas12a was also applicable for MCMV RNA detection. As we showed in our previous work (Lei et al., 2022), all the equipment can be integrated into a portable suitcase. The proposed method has great potential to enable on-site field assay of plant virus outside of laboratory.

Reporter concentration and Cas12a/crRNA cleavage reaction time affected the results of lateral flow strips, such as the intensity of test/control bands and the false positive results. It has been reported that low concentration of LF reporter easily result in false positive test band, thus decrease the detection limit (Lu et al., 2020). Sequences of 5 nt (Gootenberg et al., 2018), 6 nt (Kellner et al., 2019), or 12 nt (Bai et al., 2019) between FAM and biotin group of LF reporter have been used. In this study, we tried FAM-TTATT-biotin (5 nt) and FAM-T20-biotin (20 nt) as the ssDNA reporter of combined RT-RAA amplification and Cas12a-based detection, and found that 100 nM LF reporter (20 nt) in the final detection buffer is enough to avoid the false positive phenomena, but LF reporter with shorter sequences (5 nt) needed higher concentration (1 μM) in the final detection buffer to avoid the false positive band (Lu et al., 2020). It is important to reduce the cost of experiments, thus LF reporter (20 nt) with lower concentration is a good choice.

Several detection methods have been summarized in **Table 2**. The detection sensitivity of ELISA is dependent on the quality of antibody, and requires long incubation time (normally overnight), which is unsuitable for the rapid detection of MCMV (Uyemoto, 1980; Bernardo et al., 2021). Although RT-qPCR provides high detection sensitivity, and is the widely recognized gold standard method to detect pathogen, it requires thermos cycling and complex instrument (Zhang et al., 2011; Bernardo et al., 2021). LAMP, in spite of one of the widely used isothermal amplification techniques, requires high temperatures up to 65˚C (Chen et al., 2017), which is not well compatible with field detection requirements. In contrast, both RT-RAA and Cas12a detection systems run around 37˚C, take less time, and are more practicable for the application in rapid on-site detection. In the developed one-tube one-step RT-RAA/Cas12a lateral flow assay, the RT-RAA enzymes, dNTPs, primers, reporters, and MgAc_2_ were lyophilized and pre-stored in the bottom of a PCR tube, while Cas12a protein, crRNA, RNase inhibitor were lyophilized inside the tube lid. The total RNA extracted from maize leaves with possible infection of MCMV can be directly added to the lyophilized RT-RAA reagents, so the operation and transportation of reagents can be greatly simplified. The lateral flow assay has the advantages of visualization, portability, and simple operation (Broughton et al., 2020). The developed platform showed great potential in rapid field detection of MCMV in infected maize plants in farmland.

**Table 2:** Comparison of different detection methods for MCMV.

Method	Main instrument	Detection	Detection type	LOD	Characteristics	References
ELISA	ELISA instrument	UV spectrum	Coat Protein	0.1 μg/mL	~18h, requiring antibody	(Uyemoto, 1980)
			Coat Protein	100 pg virion protein		(Bernardo et al., 2021)
TAS-ELISA	ELISA instrument			1: 327680 (w/v, g/ml)	~21~31h, requiring antibody	(Wu et al., 2013)
RT-PCR	PCR thermocycler	Electrophoresis	Total RNA	161.4 fg	~3h, High specificity, requiring complex instruments	(Zhang et al., 2016)
	PCR thermocycler		cDNA	23.0 pg		(Jiao et al., 2019)
	PCR thermocycler		Viral RNA	10 fg		(Bernardo et al., 2021)
One-step RT-qPCR (TaqMan)	Real-time PCR	Real-time fluorescence	Total RNA	4 fg/μL total RNA (Ct value of 36.2)	~1.5h, High sensitivity, requiring complex instruments	(Zhang et al., 2011)
		Real-time fluorescence	Total RNA	1.61 fg		(Zhang et al., 2016)
		Real-time fluorescence	Viral RNA	10 fg		(Bernardo et al., 2021)
One-step RT-qPCR (SYBR Green)	Real-time PCR instrument	Real-time fluorescence	Total RNA	7.86 fg	~1.5h, High sensitivity, requiring complex instruments	(Zhang et al., 2016)
One-step RT-LAMP	60-65˚C	Electrophoresis or SYBR Green I visualization	Total RNA	2.5 pg	~1h, High specificity	(Chen et al., 2017)
RT-RPA	38˚C, water bath	Electrophoresis	cDNA	2.3 pg	~2h, High specificity	(Jiao et al., 2019)
RT-RAA/CRISPR-Cas12a visual detection	37-39 ˚C	Fluorescence visualization	cDNA	20 pg total RNA	~1.5h, Rapid, Portability, Qualitative testing	(Duan et al., 2022)
One-step RT-qPCR (TaqMan)	Real-time PCR	Real-time fluorescence	Total RNA	96 fg total RNA	~1h, High sensitivity, requiring complex instruments	This study
One-step RT-RAA/Cas12a LFD	37 ˚C, Incubator	Latera flow strips	Total RNA; Plasmid DNA containing CP gene	96 fg total RNA; 2.5 copies	<1h, High specificity and sensitivity, rapidness, portability, qualitative testing	This study
One-step RT-qRAA	39 ˚C	Real-time fluorescence	Total RNA	0.96 pg total RNA	~0.5h, High specificity, rapidness, quantification	This study

## Conclusion

In this study, a rapid and sensitive detection method based on RT-RAA/CRISPR/Cas12a system was developed for the diagnosis of maize chlorotic mottle virus (MCMV), which is responsible for a severe maize lethal necrosis disease. The limit of detection was 2.5 copies MCMV coat protein genes, and 0.96 pg of the total RNA extracted from MCMV-infected maize leaves. The developed one-tube one-step RT-RAA and CRISPR-Cas12a lateral flow assay can detect MCMV infected maize leaves at early infection time using crude extracts of total RNA from virus infected plant materials, which meets the requirement of real sample detection. The one-step RT-RAA reagents and CRISPR/Cas12a reagents can be lyophilized for easy storage and transportation of reagents, which makes this method more feasible for the filed detection. All the pipettes, reagent tubes, thermal blocks and extraction reagents are portable, which is applicable for field diagnosis of plant viral diseases.

## Data availability statement

The original contributions presented in the study are included in the article/supplementary material. Further inquiries can be directed to the corresponding authors.

## Author contributions

RL, RRK, and YJZ designed, conceived and performed the experiments. RL, HLC and XZP wrote the manuscript. ZYJ, ZXZ and ZFF validated the experiments. All authors contributed to the article and approved the submitted version.
